# Potent drug delivery enhancement of betulinic acid and NVX-207 into equine skin in vitro – a comparison between a novel oxygen flow-assisted transdermal application device and microemulsion gels

**DOI:** 10.1186/s12917-024-04064-1

**Published:** 2024-05-16

**Authors:** Paula Zscherpe, Jutta Kalbitz, Lisa A. Weber, Reinhard Paschke, Karsten Mäder, Brigitte von Rechenberg, Jessika-M. V. Cavalleri, Jessica Meißner, Karina Klein

**Affiliations:** 1 Musculoskeletal Research Unit, Vetsuisse Faculty, University of Zurich, Winterthurerstrasse 260, Zurich, 8057 Switzerland; 2 Biosolutions Halle GmbH, Weinbergweg 22, Halle (Saale), 06120 Germany; 3 Clinic for Horses, University of Veterinary Medicine Hannover, Foundation, Bünteweg 9, Hannover, 30559 Germany; 4 BioCenter, Martin Luther University Halle-Wittenberg, Weinbergweg 22, Halle (Saale), 06120 Germany; 5 Institute of Pharmacy, Faculty of Biosciences, Martin-Luther-University Halle-Wittenberg, Wolfgang-Langenbeck-Strasse 4, Halle (Saale), 06120 Germany; 6 Center for Applied Biotechnology and Molecular Medicine, Vetsuisse Faculty, University of Zurich, Winterthurerstrasse 260, Zurich, 8057 Switzerland; 7 Equine Internal Medicine, Clinical Centre for Equine Health and Research, Clinical Department for Small Animals and Horses, University of Veterinary Medicine Vienna (Vetmeduni), Veterinärplatz 1, Vienna, 1210 Austria; 8 Department of Pharmacology, Toxicology and Pharmacy, University of Veterinary Medicine Hannover, Foundation, Bünteweg 17, Hannover, 30559 Germany

**Keywords:** Equine melanoma, Betulinic acid, NVX-207, Oxygen flow-assisted transdermal application, Franz-type diffusion cell

## Abstract

**Background:**

Gray horses are predisposed to equine malignant melanoma (EMM) with advancing age. Depending on the tumor’s location and size, they can cause severe problems (e.g., defaecation, urination, feeding). A feasible therapy for EMM has not yet been established and surgical excision can be difficult depending on the location of the melanoma. Thus, an effective and safe therapy is needed. Naturally occurring betulinic acid (BA), a pentacyclic triterpene and its synthetic derivate, NVX-207 (3-acetyl-betulinic acid-2-amino-3-hydroxy-2-hydroxymethyl-propanoate) are known for their cytotoxic properties against melanomas and other tumors and have already shown good safety and tolerability in vivo. In this study, BA and NVX-207 were tested for their permeation potential into equine skin in vitro in Franz-type diffusion cell (FDC) experiments after incubation of 5 min, 30 min and 24 h, aiming to use these formulations for prospective in vivo studies as a treatment for early melanoma stages. Potent permeation was defined as reaching or exceeding the half maximal inhibitory concentrations (IC_50_) of BA or NVX-207 for equine melanoma cells in equine skin samples. The active ingredients were either dissolved in a microemulsion (ME) or in a microemulsion gel (MEG). All of the formulations were transdermally applied but the oil-in-water microemulsion was administered with a novel oxygen flow-assisted (OFA) applicator (DERMADROP TDA).

**Results:**

All tested formulations exceeded the IC_50_ values for equine melanoma cells for BA and NVX-207 in equine skin samples, independently of the incubation time NVX-207 applied with the OFA applicator showed a significant time-dependent accumulation and depot-effect in the skin after 30 min and 24 h (*P* < 0.05).

**Conclusions:**

All tested substances showed promising results. Additionally, OFA administration showed a significant accumulation of NVX-207 after 30 min and 24 h of incubation. Further in vivo trials with OFA application are recommended.

## Background

Equine malignant melanoma (EMM) in gray horses is an underestimated, progressive cutaneous neoplasm with life-threatening potential [1]. Equine melanomas have a high incidence [2] increasing with age [3–5] and representing 3.8% of neoplasms in horses [6]. They typically appear as single or multiple black nodules that arise from the skin surface, where coalescence and ulceration may occur as they progress [1, 3, 7]. Melanomas are mainly located underneath the tail, in perianal regions, lips, and eyelids [3, 7, 8]. However, they also affect parotid salivary glands, guttural pouches, paranasal sinuses, lymph nodes, cornea, and other adjacent structures [3, 7, 9–11]. The localization of the melanomas can cause various complaints such as physical obstruction of the anal sphincter, penis, prepuce, or vulva commissure, further leading to impairment of defecation, urination or breeding/mating [12, 13]. Additionally, melanomas at the head/neck region can cause respiratory obstruction, dysphagia, nerval dysfunction or difficulties bending the neck [3, 9, 10]. Dermal melanomas may be malignant from the beginning or may transform malignantly and metastasize in ageing horses [1, 11, 14–17]. A 4.6-kb duplication in intron 6 of Syntaxin 17 (STX17) is responsible for graying of hairs and development of dermal melanomas [18, 19].

Although there are several treatment options for EMM, there are only limited options particularly for advanced stages [1]. Surgical excision of melanomas seems to be a feasible, locally curative option even though it can be difficult due to the predilection sites. Recurrences at the surgery site were not described, but in approximately 50% of cases, distant melanomas showed continued growth or new melanomas developed [13, 20]. It is recommended to surgically excise the melanomas as early as possible to prevent clinical problems [13, 20]. The chemotherapeutic agent cisplatin was recorded to be efficient in studies with melanomas of various sizes, both, as an intratumoral treatment and in the form of implanted cisplatin-containing biodegradable beads [21, 22]. However, there are no suitable medical preparations available containing cisplatin in concentrations comparable to those used in the mentioned studies, as they are not approved in countries, such as Germany, Austria, and Switzerland. Additionally, chemotherapeutic agents pose risks for veterinarians, owners, horses and the environment [21, 23]. Another, newer approach for the melanoma treatment is the modification and increase of the innate immune response, cancer suppression and antitumor efficiency. It could be demonstrated that intratumorally administered plasmid DNA coding for Interleukin-12 (IL-12) [24] and IL-18 [25] induced size reduction in melanoma-bearing horses. Mählmann et al. performed intramuscular and peritumoral vaccinations with DNA vectors encoding for equine IL-12 and IL-18 and showed reduction of tumor size after three vaccinations [26].

Betulinic acid (BA) is a naturally occurring pentacyclic triterpene, widely distributed in the plant kingdom and obtained from birch tree bark. BA and its synthetic derivative, NVX-207 (3-acetyl-betulinic acid-2-amino-3-hydroxy-2-hydroxymethyl-propanoate), are known for their diverse properties, such as anti-inflammatory [27], anti-HIV [28], anthelminthic [29], immunomodulatory, antiangiogenic [30], antifibrotic and hepatoprotective effects [31, 32]. Time- and dose-dependent [23, 33] cytotoxicity of BA and NVX-207 against melanoma and various cancer cell lines from different species (e.g. human, horse, dog, mouse) has been repeatedly demonstrated in vitro and in vivo [23, 31, 33–49]. The cytotoxic mechanism of BA and its derivates is based on a CD95- and p53-independent induction of apoptosis [50, 51]. As Zuco et al. investigated antiproliferative effects of BA on human cancer cell lines and normal human cell lines which were unaffected at same concentrations, thus, the authors stated selectivity towards tumor cells over non-cancer cells [42]. Selzer et al. demonstrated melanocytes to be less susceptible to inhibition by BA than melanoma cells [34]. In cell models of different species and different cancer types, this selectivity towards tumor cells has also been observed [31, 34, 40, 42, 44, 46, 52, 53], although Weber et al. could not confirm these results in an equine cell culture model [38].

In Franz-type diffusion cell (FDC) trials, Weber et al. examined equine skin permeation of formulations containing BA and NVX-207, which exceeded the half maximal inhibitory concentrations (IC_50_) of equine melanoma cells in required skin layers [33, 38]. The authors also performed the first in vivo experiments in healthy and melanoma-bearing horses, suggesting the efficient distribution of the active ingredients BA and NVX-207 even in deeper skin layers than studied in vitro [49, 54]. As previously demonstrated in other studies [23, 32, 54], good tolerability and safety were confirmed [49, 54]. In the study of melanoma-bearing horses, in all three groups (formulations containing 1% BA, 1% NVX-207 or a placebo) reduction in tumor size was observed, whereby the effect was only significant in the BA group [49]. Considering this, and with regard to the long and intensive treatment protocol (application of the formulation twice a day for three consecutive months), the authors recommended further studies with adapted pharmaceutical formulations [49].

The skin, as the biggest organ of the body, has a natural barrier to protect itself from the environment and to prevent water loss of the body. Therefore, the utmost barrier of skin, the *stratum corneum,* must be overcome. Chemical and mechanical penetration enhancers increase the permeability of the skin for active ingredients even for hydrophilic substances or macromolecules [55]. A new medical device for oxygen flow-assisted (OFA) transdermal application (DERMADROP TDA) of active ingredients has been examined in this study. The applicator uses oxygen flow to generate fine droplets of an oil-in-water phospholipid (LP8)-microemulsion which are sprayed onto the skin surface. The LP8-microemulsion increases the permeation of the ingredients into the *stratum corneum*, and in addition, highly concentrated oxygen activates and enhances the barrier-breaking properties of the LP8-microemulsion [56]. Since it is possible to dissolve active ingredients in formulations, various active ingredients using OFA administration were tested in comparison with other delivery methods (e.g. droplets by pipette, intravenous). Increased permeation rates into skin in vitro [57] and into joints in vivo [58, 59] were demonstrated. In one study, methotrexate (MTX), a folic acid antagonist, was applied to various human skin tumors in vivo using oxygen flow [56]. Compared to the topical application of the active ingredient, a twofold higher amount of MTX was achieved within the skin when applied via oxygen flow. The tumors responded to treatment with reduction in clinical symptoms (e.g. itch, erythema, pain/pruritus, scaling), some with a reduction in histologically examined tumor nests and some with a reduction in size. As the test persons had only mild adverse reactions, the OFA administration of MTX was considered to be painless and safe [56]. In another study, a woman suffering from skin metastases from breast cancer was treated with 5% MTX OFA. During follow-up examinations, no residual cancer cells were found in a biopsy, and very good tolerability was confirmed [60].

Since EMM is a common disease in gray horses with life-threatening potential and the lack of adequate therapy options, there is an urgent need to find an effective, safe and easily-to-perform therapy. Although the IC_50_ values for BA and NVX-207 in equine skin were achieved in previously tested formulations [33, 38], the effects on melanomas size might be greater with higher concentrations of BA and NVX-207 than those recently tested in vivo [49]. Furthermore, Weber et al. recommended the investigation of different carrier substances as the formulation used was shown to have a mild skin irritant potential and sporadically caused size reduction even in the placebo group [49, 54].

Thus, this study aimed to determine whether (a) the active ingredients reach the required concentration in the skin (IC_50_ values) using FDC, (b) whether the carrier systems for BA and NVX-207 (microemulsion gel or OFA application) facilitate efficient dermal uptake of BA and NVX-207, respectively, and (c) whether a time-dependent accumulation of the active ingredients can be detected.

## Results

### Permeation rates into equine skin

The aim of this in vitro study was to determine whether the different formulations (4% BA-MEG, 10% NVX-207-MEG, 5% NVX-207-MEG, 5% NVX-207-OFA) can reach the required IC_50_ values in the dermis and whether they could be used for prospective in vivo studies. The concentrations of BA and NVX-207 were compared in at different time points in the tested skin samples without *stratum corneum (*sum of layers L2-L7, see Table 3). The concentrations of 5% NVX-207-MEG and 5% NVX-207-OFA were also determined at different time points in the single layers L2-L7 (skin depth; L2 = 11–20 µm, L3 = 21–30 µm, L4 = 31–40 µm, L5 = 41–50 µm, L6 = 51- max. 910 µm, L7 = 911 µm-end). Since the process of removing formulation from the skin sample could not be standardized, residues on the skin of the used formulations could not be ruled out. Hence, the results of the first layer (*stratum corneum*) of each experiment showed high standard deviations (data not shown) and therefore were excluded from this study.

**Fig. 1:**
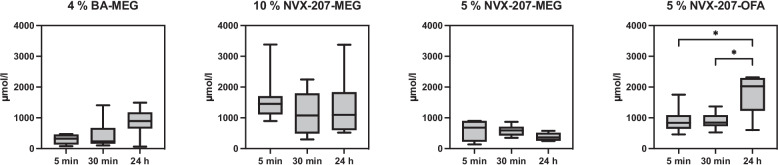
Mean concentrations and minimum and maximum values (µmol/L) in the whole skin without *stratum corneum* (sum of L2-L7) of every tested formulation. Different incubation times were shown. Concentrations of 5% NVX-207-OFA were significantly higher after 24 h compared to the concentrations after 5 and 30 min. Asterisks represent significant values (*p* < 0.05)

**Fig. 2:**
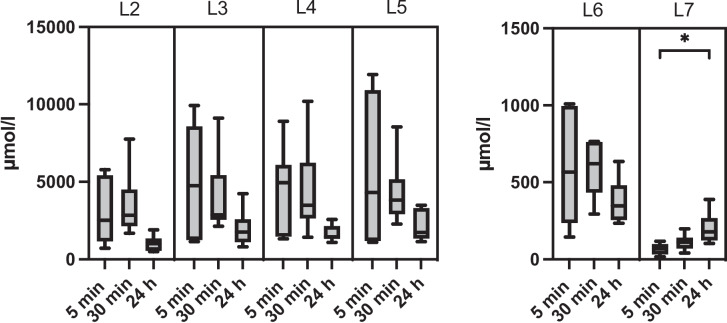
Mean concentration with minimum and maximum values (µmol/L) of 5% NVX-207-MEG in different layers (L2 = 11–20 µm, L3 = 21–30 µm, L4 = 31–40 µm, L5 = 41–50 µm, L6 = 51- max. 910 µm, L7 = 911 µm-end) after different incubation times. For better visualization, L6 and L7 were presented with a different scale from 0 to 1500 µmol/l. Concentrations of 5% NVX-207-MEG in layer 7 after 24 h of incubation were significantly higher than after 5 min of incubation. Asterisks represent significant values (*p* < 0.05)

**Fig. 3:**
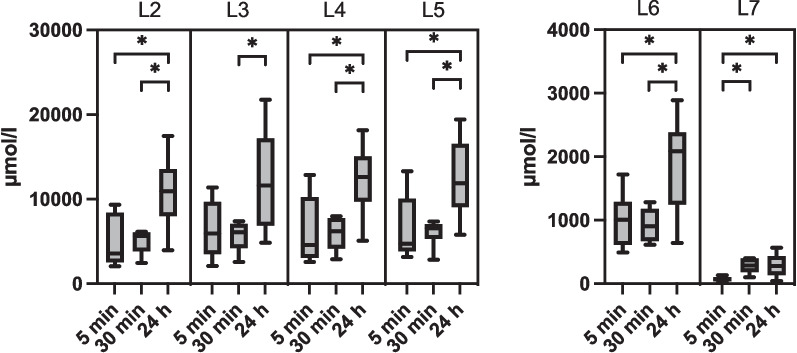
Mean concentration with minimum and maximum values (µmol/L) of 5% NVX-207-OFA in different layers (L2 = 11–20 µm, L3 = 21–30 µm, L4 = 31–40 µm, L5 = 41–50 µm, L6 = 51- max. 910 µm, L7 = 911 µm-end) after different incubation times. For better visualization, L6 and L7 were presented with a different scale from 0 to 4000 µmol/l. 5% NVX-207-OFA showed significantly higher (*p* < 0.05) concentrations of NVX-207 in the layers 2,4,5 and 6 after 24 h of incubation compared to 5 and 30 min of incubation. The concentrations of NVX-207 in layer 3 after 24 h were significantly higher (*p* < 0.05) compared to the 30 min values, and also in layer 7 after 24 h compared to the concentrations after 5 min incubation. In layer 7, after 30 min a significant (*p* < 0.05) increase was also detectable compared to the 5 min incubation values. Asterisks represent significant values (*p* < 0.05)

4% BA-MEG, 10% NVX-207-MEG, and 5% NVX-207-OFA indicated a time-dependent increase of BA and NVX-207 concentrations in the whole skin without *stratum corneum*, but only the 24-h (h) values of 5% NVX-207-OFA were significantly higher than the ones after 5 and 30 min (min) of incubation (*p* < 0.05; Fig. 1). 5% NVX-207-MEG showed a time-dependent decrease in concentration with the lowest values after 24 h. Comparing the values of the different layers time-dependently, 5% NVX-207-MEG showed decreasing values after longer incubation periods, although the 24 h layer 7 was significantly (*p* < 0.05) higher than the 5 min values (Fig. 2). 5% NVX-207-OFA showed significantly higher (*p* < 0.05) concentrations of NVX-207 in the layers 2,4,5 and 6 after 24 h of incubation compared to 5 and 30 min of incubation. The concentrations of NVX-207 in layer 3 after 24 h were significantly higher (*p* < 0.05) compared to the 30 min values, and also in layer 7 after 24 h compared to the concentrations after 5 min of incubation. In layer 7, after 30 min a significant (*p* < 0.05) increase was also detectable compared to the 5 min incubation values (Fig. 3). Independently of incubation time or layer one horse always showed the lowest permeation rates. Since the thickness of the L2-L5 and L6 and L7 differed (L2-L7: 10 µm, L6: max. 860 µm, L7: max. 600 µm), a dilution effect was observed so that the total concentrations (µmol/L) were lower than in L2-L5.

### Acceptor media

The acceptor medium is in direct contact with the underside of the skin sample in the acceptor chamber. No active ingredient was found in the acceptor media treated with 10% NVX-207-MEG, 4% BA-MEG or 5% NVX-207-OFA, respectively. Only one acceptor medium of the experiment with 5% NVX-207-MEG incubated for 24 h contained 1.7% of the NVX-207 used.

### Histological examination

**Fig. 4:**
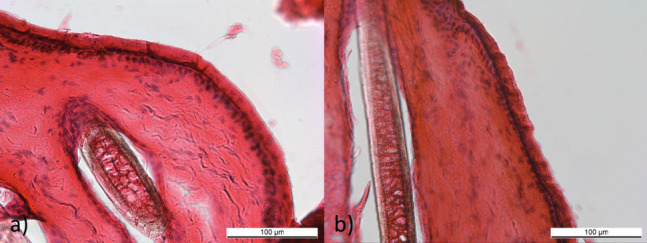
Histological section of equine skin treated with 5% NVX-207-OFA, applied either with a pipette (control, **a**) or with the OFA applicator (**b**). No microscopical alterations of the stratum corneum were noticed. Samples were cut transversally into 10 µm thick layers and stained with HE. 20-fold magnification, the bar represents 100 µm

Skin treated with 5% NVX-207-OFA applied with the OFA-applicator or pipette was examined for microscopical alterations in the *stratum corneum* as it represents the natural skin barrier (Fig. 4). For this purpose, the integrity of the *stratum corneum* was assessed. No destruction of the *stratum corneum* was observed after the treatment with the OFA applicator or the pipetted solution.

## Discussion

This study aimed to investigate if (a) BA and NVX-207 used as treatment against EMM could reach required concentrations in dermal skin layers and, if (b) the different carriers (MEG vs. OFA) showed different efficacy in permeation of the skin and whether (c) an accumulation in the skin can be achieved.

In the present study, all tested formulations permeated the *stratum corneum* and the active ingredients reached deeper dermal layers after every incubation time. 5% NVX-207-OFA showed a significant time-dependent accumulation of NVX-207 after 24 h as well as after 30 min for the deepest layer (L7). After 24 h of incubation, 5% NVX-207-MEG also had significantly higher NVX-207 values in L7. Weber et al. determined IC_50_ values of BA and NVX-207 for different equine melanoma cell lines after 24 h and other time points [33, 38]. To the authors’ knowledge, there were no IC_50_ values for BA and NVX-207 for 5 min and 30 min incubation periods published, so just the 24 h concentrations (Table 1) could be compared with published data. In the current study, all of the 24 h incubated skin samples reached and exceeded the reported IC_50_ values (Table 2). Since every sample exceeded the necessary amounts, all of the tested formulations and carrier systems seemed to be feasible for further in vivo investigations. Interestingly, 5% NVX-207-MEG concentrations in the skin decreased in a time-dependent manner though this was not detectable for the 10% NVX-207-MEG. Internal controls (data not shown) demonstrated, that dissolved NVX-207 was thermolabile as it tended to degrade at room temperature. Thus, the NVX-207 in formulation was stored at fridge temperature (approximately 4 °C). A possible reason for the decreasing values of 5% NVX-207-MEG could be changes in the chemical structure of NVX-207 during the experiment leading to detection difficulties in HPLC. Significantly higher (*P* < 0.05) NVX-207 values could be demonstrated after 24 h incubation for all 5% NVX-207-OFA treated layers. In L7 (deepest layer; 911 µm until the sample is completely depleted) after 30 min of incubation, NVX-207 increased significantly (*p* < 0.05) compared to the 5 min values. These results represented an accumulation of 5% NVX-207 in a time-dependent manner and indicated a depot effect of the oxygen flow-assisted transdermal application. However, one horse of the 5% NVX-207-OFA experiments, which had always the lowest permeation rates, accumulated significantly (*p* > 0.05). It was a 24 years-old German Riding Pony euthanized because of a small intestine strangulation. As no unique characteristics for this horse could be identified and this sample was processed like all the other specimens, no specific reason for the lower rates could be investigated. Time-dependent accumulation and depot formation following OFA treatment were demonstrated in the present experiment as well as in other studies before [56–59]. Sidler et al. performed in vivo studies in sheep with cartilage defects in the knee treated with oxygen flow-assisted transdermal or intravenous application of the non-steroidal anti-inflammatory drug carprofen. Higher, cumulated carprofen concentrations in the synovia after OFA administration were demonstrated as well as a better histological remodeling of the joint defects compared with intravenous administration of carprofen [58, 59]. The findings of the present study underline the accumulating effects of the OFA application as a significant time-dependent increase could be demonstrated. However, the findings of Sidler et al. indicate, how OFA-applied formulations can behave in vivo. Elksnat et al. investigated in an in vitro FDC experiment the permeation potential of oxygen flow-assisted transdermal application [57]. Various active ingredients with varying molecular weights, lipophilicities and melting points (e.g., diclofenac, enrofloxacin, salicylic acid) were applied to porcine skin using FDC either with oxygen flow or an equivalent amount with a pipette. All tested formulations had time-dependent higher concentrations when applied with oxygen flow [57]. The authors stated that active ingredients with lower molecular weights permeated more efficiently than larger molecules. In addition to the active ingredients investigated by Elsknat et al., carprofen and methotrexate were also applied with oxygen flow in other studies [56, 58, 59]. All of the tested active ingredients had lower molecular weights than NVX-207 (MW: 601,9 g/mol) [35]. NVX-207 could easily be administered with the OFA applicator and reached high amounts in deeper skin layers in the present study. Even 30 min after OFA treatment, a significant accumulation of NVX-207 was observed in the deepest tested layer (L7). In a pilot study from Lebas et al., human probands suffering from different skin cancers, were treated with OFA-applied methotrexate, a folic acid antagonist [56]. Compared with the MTX formulation administered transdermally without oxygen, OFA administration of MTX resulted in twice the MTX concentration in the skin and improved clinical and histologic regression in epidermal and superficial dermal layers. The authors hypothesized that MTX with its high molecular weight (454,4 g/mol) and hydrophilic properties was caught in the skin and could not be removed by blood circulation. Thus, a depot effect of the active ingredient might be possible. This hypothesis is of great interest for the present study, because, as mentioned before, NVX-207 with its high molecular weight was able permeate the *stratum corneum* and to accumulate in dermal layers in the FDC experiment. Further investigations on how NVX-207 behaves in a living organism after OFA application should be the purpose for following studies.

**Table 1:** IC_50_ values for BA and NVX-207 for equine melanoma cells after 24 h of incubation, published by Weber et al. [33, 38], determined with cytotoxic MTS assay, antiproliferative crystal violet staining (CVS) assay; Data represent mean IC_50_ values (μmol/L) with 95% confidence interval in parentheses

BA			NVX-207		
Cell type	MTS	CVS	Cell type	MTS	CVS
eRGO1	22.8 (− 3–48)	25.9 (20–32)	eRGO1	7 (4–15)	5 (3–7)
MelDuWi	34.6 (24–45)	49.2 (31–67)	MelDuWi	18 (15–21)	16 (11–21)

**Table 2:** Presentation of the x-fold exceedance of the required IC_50_ values for BA and NVX-207 (IC_50_ values determined by Weber et al. with cytotoxic MTS assay, antiproliferative crystal violet staining (CVS) assay, Table 1 [33, 38]) after 24 h of incubation (range of minimum to maximum was presented)

BA			NVX-207		
4% BA-MEG	2—65	1—58	10% NVX-207-MEG	29—482	32—675
			5% NVX-207-MEG	14—82	15—115
			5% NVX-207-OFA	33—331	37—464

In the present study, the histological examination of the *stratum corneum* was performed to assess if the treatment with the OFA applicator alters the integrity of the *stratum corneum* and other upper skin structures. The *stratum corneum* was still intact after treatment with the OFA applicator and therefore no hazard for the dermis or deeper structures is expected. Elksnat et al. also examined the integrity of porcine *stratum corneum* after treatment with oxygen flow [57]. As demonstrated in the current study, no changes in the *stratum corneum* were detected. It should be mentioned that the histological examination should only be considered as exemplary, as although 10 slices were examined, these were only from one animal. Further studies on this topic are recommended.

The results of the present study promoted the potent permeation properties of both the MEG and the OFA application system for BA and NVX-207 in higher concentrations than previously tested. Microemulsions are expected to be superior to creams tested in previous studies [49, 54] with respect to solubilization and penetration properties. Thus, the tested formulations and OFA application are worthwhile for further investigations in vivo.

For technical reasons and due to standardization difficulties, skin affected by EMM could not be used to perform the FDC experiments. For the same reason, Weber et al. also used equine skin from the lateral thorax since Mills et al. showed that topically applied hydrocortisone had similar permeation rates in thoracic and groin skin [33, 61]. Therefore, permeation rates from the ventral side of the tail were expected to be comparable to those from the thoracic or groin skin. This is a limitation of the performed in vitro FDC model as well as the missing, physiologic blood circulation. Additionally, the tested skin was harvested from various horses with different breed, sex and age which might explain the variabilities in the concentrations of BA or NVX-207. As melanomas affect all equine breeds, sex and ages, the authors wanted to see how the formulations permeate in the skin of various horses. Due to technical reasons (e.g., too long storage times, too small harvested skin samples), it was not possible to test all formulations on the same horses which limited the results of this study. Regarding the concentration of BA and NVX-207 in the single slices or pooled samples, the samples were not totally uniform, as the individual thicknesses of equine skin were never completely equal, even though each sample was processed in the cryostat in the same way. Furthermore, repeated freezing and thawing of the skin samples (e.g., for dermatomization, FDC experiment, cryostat) is likely to alter the skin and thus the permeation properties, since Ahlstrom et al. investigated hydrocortisone permeation profiles of canine skin [62]. They demonstrated that the extent of permeation increased with freezing but not the shape of the profiles itself, but for technical reasons this cannot be carried out in any other way in the present study.

## Conclusion

All formulations demonstrated a potent permeation of the *stratum corneum* into deeper dermal layers exceeding the required IC_50_ values indicating promising results. Therefore, further studies to investigate the formulations’ potential for topical treatment of equine melanomas are recommended.

The OFA application system was a feasible and promising drug delivery enhancer being able to build an active ingredient depot in the skin including time-dependent accumulation. Thus, it may be interesting for further studies for safe and non-invasive topical treatment.

## Methods

### Skin samples

For the permeation tests, skin samples of horses euthanized for reasons unrelated to this study were used (Clinic for Horses, University of Veterinary Medicine Hannover, Foundation, Hannover). A total of 24 horses were included in the study. For each formulation, the skin of six different horses was analyzed. As described in detail in Table 3, the horses were of different sexes (6 mares, 11 geldings, 7 unknown), breeds (8 warmbloods, 3 Icelandic horses, 2 German Riding Ponies, 2 heavy warmbloods, 1 Arabian, 1 Dulmener Horse, 7 unknown) and ages (ranging from 3 to 25 years with a mean of 15 years). Skin samples were harvested by the Institute of Pathology (University of Veterinary Medicine Hannover, Foundation) and dissected from the lateral thorax and stored at -20 °C for up to a maximum of five weeks until further processing. The skin was defrosted overnight at room temperature and the hair was carefully clipped to a length of 0.5 mm. After that, the samples were examined macroscopically for superficial lesions or other abnormalities. Altered skin was excluded from this study. Skin sections were cut with an electrical dermatome (Zimmer, Eschbach, Germany) to a depth of at least 580 µm and a maximum of 1710 µm (mean: 963 µm, SD: 271 µm). Specimens were cut into size (approximately 3 × 3 cm; 9 cm^2^ surface) with a scalpel, wrapped into aluminum foil, and frozen at – 20 °C until the start of the experiment. The storage duration from skin preparation until test day was a maximum of 43 days.

**Table 3:** Detailed information about the skin’s origin depending on the tested substance

Tested drug formulation	Breed	Sex	Mean age in years (range min–max)
4% BA-MEG	2 Hanoverian Warmbloods, 1 German Sport Horse, 1 East Frisian Horse/Old-Oldenburger, 1 Arabian, 1 unknown	3 geldings, 2 mares, 1 unknown	15 (8–21)
10% NVX-207-MEG	3 Icelandic Horses, 1 Hanoverian Warmblood, 1 German Riding Pony, 1 unknown	4 geldings, 1 mare, 1 unknown	16 (4–25)
5% NVX-207-MEG	3 Hanoverian Warmbloods, 1 Dulmener Horse, 2 unknown	3 geldings, 1 mare, 2 unknown	18 (13–24)
5% NVX-207-OFA	1 Oldenburger Warmblood, 1 Saxon Heavy Warmblood, 1 German Riding Pony, 3 unknwon	2 mares, 1 gelding, 3 unknown	11 (3–23)

## Oxygen flow-assisted transdermal application system

**Fig. 5:**
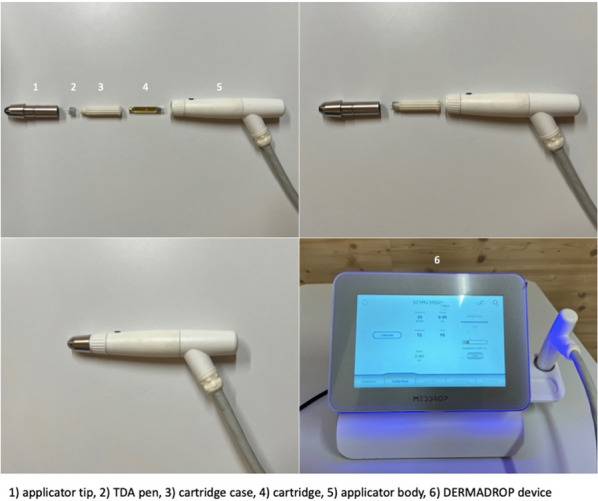
Oxygen flow-assisted transdermal application system (DERMADROP TDA)

**Fig. 6:**
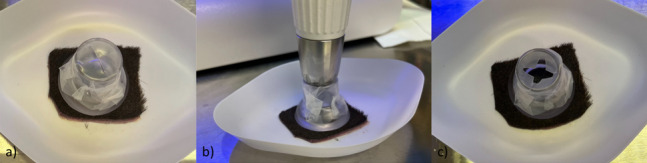
Administration of 5% NVX-207-OFA with the OFA-application system for the FDC experiment: before the experiment (**a**), during the application (**b**), and after the experiment (**c**). Afterwards, the skin was mounted on the FDC

The oxygen flow-assisted (OFA) transdermal application system (DERMADROP TDA; Meddrop BioMedical Technologies GmbH, Hamburg, Germany) consisted of an oxygen supply and an applicator that, according to the manufacturer, delivered the microemulsion to the skin surface at 1 bar. In detail, at an average rate of 98%, medical oxygen flowed through a valve inside the applicator, reducing the pressure, and flowed to a cartridge containing the medical formulation as a microemulsion. Taking advantage of the venturi effect (fluid pressure decreases in constricted areas) the oxygen propelled the formulation and atomized it into droplets with a size of nanometers. Through the so-called transdermal application pen (TDA-pen, Insupen32G, 0,23 × 8 mm, Pikdare S.p.A., Casnate con Bernate, Italy), the droplets were applied onto the skin (Fig. 5, previously published from the same research group [57]) with the applicator tip at a 90° angle to the skin surface with a distance equal to the height of the donor chamber of the FDC (approximately 20 mm). The donor chamber was covered using parafilm with a cross-shaped hole for the applicator tip. It was mounted onto the skin while the formulation was applied to prevent the substance from spreading in surrounding areas (Fig. 6).

## Formulations

Four different formulations were tested for their permeation rates. BA or NVX-207 were dissolved in a) microemulsion gels (containing either 4% BA or 5% NVX-207 or 10% NVX-207) and b) a LP8-microemulsion (containing 5% NVX-207), applied with the oxygen flow-assisted transdermal applicator. BioSolutions Halle GmbH, Halle, Germany, provided BA and NVX-207. The microemulsion gels were provided by Martin-Luther University Halle-Wittenberg, Institute for Pharmacy, Halle, Germany. The microemulsion gels with BA contained BA, NMP, Macrogol 15 Hydroxystearate (Ph. Eur.), middle chain triglycerides (MCT) (Ph. Eur.), middle chain partial glycerides (Ph. Eur.). The microemulsion gels with NVX-207 contained NVX-207, ethylcellulose (Ph. Eur.), Macrogol 15 Hydroxystearate (Ph. Eur.), middle chain triglycerides (MCT) (Ph. Eur.), middle chain partial glycerides (Ph. Eur.). Exact quantities or concentrations have not been published in this publication. Meddrop BioMedical Technologies GmbH supplied the LP8-microemulsion (consisting of PEG-8 Caprylic/Capric Glycerides, Diethylene Glycol Monoethyl Ether, Polyglyceryl-6 Dioleate, Phosphatidylcholine, Propylene Glycol, Alcohol Denat., Aqua, Tocopherol) in which the performing scientist (PZ) dissolved the NVX-207 with Isopropanol and water Ph. Eur.. Due to the low solubility of BA, it was not possible to dissolve in the oil-in-water microemulsion for OFA application. The concentration of 4% BA-MEG was selected to have a security window as concentrations of BA exceeding 5% did show storage dependent precipitation.

## Skin permeation tests

The permeation tests were performed using the Franz-type diffusion cells (PermeGear, Riegelsville, USA, and Gauer Glas, Püttlingen, Germany) with an acceptor chamber volume of approximately 12 ml and a diffusion area of 1.77 cm^2^. The previously published test procedure [33, 38] was partly adapted as follows:

The acceptor chamber was filled with phosphate-buffered saline (PBS, pH 7.4, containing 0.2 g KCl, 8.0 g NaCl, 0.2 g KH2PO4, 1.44 g Na2HPO4 × 2H2O per liter deionized water, obtained from Merck KGaA, Darmstadt, Germany) and 1% bovine serum albumin (obtained from Merck KGaA, Darmstadt, Germany), which was magnetically stirred at 500 rpm and heated to 34 °C to simulate the physiological skin surface temperature.

On the day of the experiment, the skin samples were thawed at room temperature and inserted into PBS for 30 min for equal hydration. Immediately after this, the samples were gently dried with paper tissue. Further test execution depended on the carrier substance:

- Microemulsion gels (containing either 4% BA or 10% NVX-207 or 5% NVX-207): 20 mg of the microemulsion gel was applied on the skin’s upper surface (*stratum corneum*) and gently distributed onto the diffusion area with a spatula. Then the skin was mounted on the FDC and the donor chamber and the sampling port was covered with parafilm. Only the samples, which were incubated for five min, were not covered with parafilm. Regarding the residues on the spatula used, the spatula was weighed after distribution. The difference between the residues on the spatula and the amount of formulation on the sample was taken into account in further calculations.
- OFA transdermal application: 43 μl of the formulation was applied on the skin surface in the diffusion area, as previously described. Further procedure corresponded to that of the microemulsion gel specimens.

After 5 min, 30 min, or 24 h the samples were removed from FDC, and the skin surface as well as every surface that was in contact with the formulation (donor chamber, scalpel, cutting board) were cleaned from the remaining formulation with a cotton swap. The diffusion area was cut out with a scalpel and frozen at -20 °C together with the acceptor media and the cotton swaps until further processing. For the 5% NVX-207-OFA samples, the donor chambers for the application were cleaned with cotton swaps and a cleaning solution, which were stored at -20 °C as well as the parafilms from the application. The cleaning solutions consisted either of methanol (MeOH, AppliChem GmbH, Darmstadt, Germany) for the BA specimens or 80% methanol and 0,1% formic acid (HCOOH, AppliChem GmbH, Darmstadt, Germany) for the NVX-207 samples.

## Sample preparation for quantitative analysis

To determine the amounts of the different active ingredients in the skin, the samples were cut parallel to the skin surface with a cryostat (CyroStar™ NX70 Cryostat, Thermofisher, Darmstadt, Germany) that was capable of cutting slices up to a depth of 0.5 µm. The angle of the cryostat’s specimen holder was adjusted so that the blade cut parallely to the skin surface. The samples were fixed on a tissue freezing medium (Tissue-Tek®, Sakura Finetek Europe B.V., Alphen aan den Rijn, Netherlands) with the *stratum corneum* side upmost, which was cut into a 10 µm thick slice (layer 1, L1) and stored separately for every sample of every formulation tested. Based on the data of Bizley et al. [63] and since it could not be guaranteed that the skin surfaces were completely flat, 10 µm seemed to be an appropriate thickness to cut the stratum corneum without inadvertently cutting deeper. Different slicing protocols were performed for the tested formulations (Table 4):

- 4% BA-MEG: after the *stratum corneum* (L1), 20 µm thick slices were cut and pooled in one vial until a depth of a maximum 910 µm was reached and if necessary, 20 µm thick slices were pooled until the sample was depleted.
- 10% NVX-207-MEG: after the *stratum corneum*, 20 µm thick slices were cut and pooled in one vial until a maximum depth of 910 µm was reached.

**Table 4:** Different cutting protocols for the cryostat for every tested formulation. Representing the different layers with their respective depth

Layers	4% BA-MEG	10% NVX-207-MEG	5% NVX-207-MEG	5% NVX-207-OFA
L1	0–10 µm	0–10 µm	0–10 µm	0–10 µm
L2	11- max. 910 µm (pooled)	11- max. 910 µm (pooled)	11–20 µm	11–20 µm
L3			21–30 µm	21–30 µm
L4			31–40 µm	31–40 µm
L5			41–50 µm	41–50 µm
L6			51- max. 910 µm (pooled)	51- max. 910 µm (pooled)
L7	possibly 911 µm-end (pooled)		possibly 911 µm-end (pooled)	possibly 911 µm-end (pooled)
L8	∑ L2—L7	∑ L2—L7	∑ L2—L7	∑ L2—L7

To determine the permeation in the upper skin layers, skin treated with 5% NVX-207-MEG and 5% NVX-207-OFA was cut according to the following protocol: after the stratum corneum (L1), 10 µm thick slices were cut and stored separately until a depth of 50 µm (L2, L3, L4, L5), then 20 µm thick slices were cut until a maximum depth of 910 µm (L6) and if necessary, 20 µm thick slices were pooled until the specimen was depleted (L7).

Between each 10 µm thick slice and each pooled sample, the blade was cleaned with the respective cleaning solution. The skin samples as well as the cleaning tissues were stored at -20 °C until analytic high-performance liquid chromatography (HPLC).

## Quantity of skin samples

The quantity of skin samples for every formulation and every incubation time was six (Tables 5 and 6). For 10% NVX-207-MEG additional four samples (5 min incubation), three samples (30 min incubation), and two samples (24 h incubation) were cut. The quantity of samples of layer 7 of the experiments with 5% NVX-207-MEG and 5.

**Table 5:** Quantity of skin samples

Formulation	5 min	30 min	24 h
4% BA-MEG	6	6	6
10% NVX-207-MEG	11	9	8
5% NVX-207-MEG	6	6	6
5% NVX-207-OFA	6	6	6

**Table 6:** Quantity of skin samples for the different layers of 5% NVX-207-MEG and 5% NVX-207-OFA

	5% NVX-207-MEG			5% NVX-207-OFA		
Layer	5 min	30 min	24 h	5 min	30 min	24 h
L2	6	6	6	6	6	6
L3	6	6	6	6	6	6
L4	6	6	6	6	6	6
L5	6	6	6	6	6	6
L6	6	6	6	6	6	6
L7	5	6	6	6	5	6

% NVX-207-MEG was occasionally lower because the sample was depleted and no skin was left.

## Quantitative analysis of the skin with high-performance liquid chromatography (HPLC)

The quantitative analysis of the skin samples and additional specimens (e.g., cleaning tissues) was performed with reverse phase analysis as described before by Weber et al. Briefly, for BA Agilent 1100 system (Agilent, Waldbronn, Germany) on a Kinetex column (5 μm, C18, 100 Å, 250 × 4.6 mm; Phenomenex, Torrance, US) was used at 35 °C developing with acetonitrile:water (0.1% HCOOH) 4:1 (v/v) at 2.5 mL/min [38]. NVX-207 was quantified with an Agilent 1100 system (Agilent) on a Luna® Omega column (3 μm, PS C18, 100 Å, 150 × 4.6 mm; Phenomenex, Torrance, US) at 30 °C utilizing a gradient method with acetonitrile (0.1% HCOOH)(A): water (0.1% HCOOH)(B) at 1.1 ml/min (from 60 to 10% B within 7.50 min) [33]. For both ingredients, the diode array detector was set at 200 nm. The amount of BA or NVX.207 was converted into the volume of the slice or the pooled samples so that the concentration of active ingredient per slice or pooled sample could be specified in µmol/L. The volume of the slices was calculated using the diameter of the sample (1.77 cm^2^) and the thickness of the slices respectively the pooled slices.

## Histological examination

To determine the effect of the OFA application on the skin, equine skin was prepared for the FDC experiment as previously described. The implementation was similar to that recently published [57]. 43 µl of 5% NVX-207-OFA was applied to one skin sample using the OFA applicator and an equivalent amount of the substance was applied to another skin sample from the same horse with a pipette. The skin was frozen until further processing. Then, the skin was fixed with tissue freezing medium and cut transversally to the skin surface in 10 µm thick slices with the cryostat at -20 °C. The skin was stained with hematoxylin and eosin (HE) after the protocol of Stahl et al. [64]. For each group (OFA and control), ten different sections were selected in a 20-fold magnification. The histological examinations of the *stratum corneum* were performed with the light microscope Leica DM6000B (Leica Microsystems Inc., Deerfield, Illinois, USA) and the software Imagic ims Client (Imagic Bildverarbeitung AG, Glattbrugg, Switzerland).

## Statistical analysis

A Univariate Analysis of Variance (ANOVA) was used to calculate statistically significant overall differences between concentrations of the active ingredients in the different layers (all: L8; for 5% NVX-207-MEG and 5% NVX-207-OFA L2-L7 solitary) after the respective incubation times to determine time-dependent effects.

Posthoc tests according to Bonferroni were used to assess individual differences between groups. Statistical analysis was performed with IBM SPSS Statistics (Version 28, International Business Corporation IBM, New York, USA) and *p*-Values < 0.05 were considered statistically significant. The box and whisker plots were designed with GraphPad Prism 9.5.1 (GraphPad Software Inc., San Diego, CA, USA).

## Data Availability

The datasets analyzed during the current study are available from the corresponding author on reasonable request.

## Abbreviations

BA: Betulinic acid
EMM: Equine malignant melanoma
FDC: Franz-type diffusion cell
HE: Hematoxylin eosin staining
HPLC: High-performance liquid chromatography
L2-8: Layers 2–8
LP3: Fore-emulsion with phospholipids
LP8: Fore-emulsion with phospholipids
H: Hour(s)
IC_50_: Half maximal inhibitory concentrations
NVX-207: 3-Acetyl-betulinic acid-2-amino-3-hydroxy-2-hydroxymethyl-propanoate
ME: Microemulsion
MEG: Microemulsion gel
Min: Minute(s)
MF: Mycosis fungoides
MTS: CellTiter 96® AQueous One Solution Cell Proliferation Assay (Promega)
MTX: Methotrexate
MW: Molecular weight
OFA: Oxygen flow-assisted
PBS: Phosphate-buffered saline
PEG: Polyethylene glycol
Ph. Eur: European Pharmacopoeia
ROS: Reactive oxygen species
SCD-1: Stearoyl-CoA desaturase
SD: Standard deviation
STX17: Syntaxin 17
Tab.: Table
TDA: Transdermal application
